# RaNet: a residual attention network for accurate prostate segmentation in T2-weighted MRI

**DOI:** 10.3389/fmed.2025.1589707

**Published:** 2025-06-26

**Authors:** Muhammad Arshad, Chengliang Wang, Muhammad Wajeeh Us Sima, Jamshed Ali Shaikh, Salem Alkhalaf, Fahad Alturise

**Affiliations:** ^1^College of Computer Science, Chongqing University, Shapingba, Chongqing, China; ^2^Department of Computer Engineering, College of Computer, Qassim University, Buraydah, Saudi Arabia; ^3^Department of Cybersecurity, College of Computer, Qassim University, Buraydah, Saudi Arabia

**Keywords:** RaNet, deep learning, refine feature selection, medical image segmentation, prostate cancer, feature fusion

## Abstract

Accurate segmentation of the prostate in T2-weighted MRI is critical for effective prostate diagnosis and treatment planning. Existing methods often struggle with the complex textures and subtle variations in the prostate. To address these challenges, we propose RaNet (Residual Attention Network), a novel framework based on ResNet50, incorporating three key modules: the DilatedContextNet (DCNet) encoder, the Multi-Scale Attention Fusion (MSAF), and the Feature Fusion Module (FFM). The encoder leverages residual connections to extract hierarchical features, capturing both fine-grained details and multi-scale patterns in the prostate. The MSAF enhances segmentation by dynamically focusing on key regions, refining feature selection and minimizing errors, while the FFM optimizes the handling of spatial hierarchies and varying object sizes, improving boundary delineation. The decoder mirrors the encoder's structure, using deconvolutional layers and skip connections to retain essential spatial details. We evaluated RaNet on a prostate MRI dataset PROMISE12 and ProstateX , achieving a DSC of 98.61 and 96.57 respectively. RaNet also demonstrated robustness to imaging artifacts and MRI protocol variability, confirming its applicability across diverse clinical scenarios. With a balance of segmentation accuracy and computational efficiency, RaNet is well suited for real-time clinical use, offering a powerful tool for precise delineation and enhanced prostate diagnostics.

## 1 Introduction

Medical image segmentation is vital for disease diagnosis, lesion localization, treatment planning, and surgical navigation. Traditional methods, requiring manual feature extraction, are often computationally expensive and lack flexibility across different clinical scenarios. In contrast, deep learning, especially convolutional neural networks (CNNs), has revolutionized segmentation by enabling end-to-end learning directly from data, eliminating the need for manual feature design (1–3). Among the deep learning architectures, U-Net (4) has gained prominence due to its efficient encoder-decoder structure, which integrates semantic features for precise segmentation. Recent advancements, including dilated convolutions (5), attention mechanisms (6), and multi-scale feature extraction (7), have further enhanced U-Net's capabilities, addressing challenges like ambiguous boundaries and complex anatomical structures. Despite these advancements, CNNs still struggle with modeling long-range spatial dependencies, which are critical for resolving complex structures, especially in medical images with subtle contrast variations and adjacent tissue influences. This limitation has prompted the exploration of transformer-based models (8, 9), which excel at capturing global context, improving segmentation accuracy. However, incorporating long-range dependencies remains an ongoing challenge for segmentation tasks where contextual information is essential.

Prostate cancer is one of the leading causes of cancer-related deaths in men, making accurate prostate segmentation on T2-weighted magnetic resonance imaging crucial for diagnosis, treatment planning, and disease monitoring. While manual segmentation by radiologists is the gold standard, it is time-consuming and prone to inter-observer variability. Automated prostate segmentation has proven difficult due to complex anatomical textures, subtle contrast differences, and imaging artifacts such as motion blur and inhomogeneity (10, 11). Despite advancements in MRI technology, automated prostate segmentation remains challenging due to the region's complex anatomical textures, subtle contrast variations with adjacent tissues, and imaging artifacts such as motion blur or inhomogeneity. While machine learning (ML) and deep learning (DL) methods have shown promise, conventional models often fail to capture the PZ's intricate patterns (12). Standard convolutional neural networks (CNNs), constrained by fixed-size kernels, struggle to model long-range spatial dependencies essential for resolving ambiguous boundaries (13). Furthermore, variability in MRI acquisition protocols, patient anatomy, and artifact profiles limits the model's robustness to MRI artifacts and generalizability across different clinical settings, as demonstrated by the experimental results in later sections (14). Despite significant advancements in medical image segmentation, several challenges remain. Existing segmentation models struggle to capture complex patterns and subtle variations in medical images, particularly in challenging regions like the prostate. Additionally, they often fail to effectively focus on relevant regions and refine feature maps at multiple scales, leading to reduced accuracy. Variability in semantic and spatial information from multiple decoder layers also limits segmentation performance.

To address these limitations, we propose RaNet, a novel ResNet50-based framework enhanced with dynamic attention mechanisms. By integrating attention modules into skip connections, RaNet adaptively prioritizes relevant regions in T2-weighted MRI, suppressing noise while refining feature extraction for precise prostate localization. This approach aims to overcome the shortcomings of fixed-receptive-field CNNs and variability-induced performance degradation, offering a robust solution for accurate and generalizable prostate segmentation.

The key contributions of this paper are as follows:

- Introduce a robust encoder-decoder architecture that utilizes residual connections to effectively extract hierarchical features while preserving spatial details, allowing the model to capture complex patterns and subtle variations in medical images, particularly in the prostate.
- Propose a novel MSAF that acts as an attention mechanism at skip connections, enabling the model to focus on relevant regions and refine feature maps at multiple scales. This refinement improves segmentation accuracy and reduces errors.
- Integrate a FFM that fuses outputs from multiple decoder layers using bilinear upsampling. This module effectively combines both semantic and spatial information, leading to more accurate and robust segmentation results.
- Extensive experiments on a large MRI dataset demonstrate the superior performance of the proposed model, achieving a Dice similarity coefficient of 0.92. The model also reduces false positive and false negative rates, outperforming conventional segmentation methods.

This article is structured into several key sections that address our research on prostate segmentation. Section 2 reviews related work, highlighting various modular approaches. Section 3 outlines our automated segmentation framework, detailing its innovative components. Section 4 describes the dataset, implementation details, and evaluation metrics, Section 5 along with experimental results supported by ablation studies and explain the comparative studies with other state-of-the-art methodologies. In Section 6, critically discuss the findings and their implications within the existing literature. Finally, Section 7 summarizes the key insights and suggests directions for future research.

## 2 Related work

Prostate segmentation, particularly in T2-weighted MRI, presents significant challenges due to complex anatomical textures, subtle contrast variations, and the influence of surrounding tissues. In recent years, various advancements in deep learning architectures have been proposed to address these challenges and improve segmentation accuracy. The related work in this area is categorized into three key sections: encoder architectures, attention mechanisms in skip connections, and multi-scale feature fusion. These approaches have contributed significantly to enhancing the robustness and performance of prostate segmentation models, as outlined in the sections below.

### 2.1 Encoder architectures for medical imaging

The success of deep learning in medical segmentation is anchored in encoder-decoder architectures such as U-Net (4), which employs a symmetric structure to hierarchically extract features through its contracting path (encoder) and reconstruct precise segmentations via its expanding path (decoder), bridged by skip connections to retain spatial details. Building on this foundation, He et al. (15) introduced ResNet50, which addresses vanishing gradients in deep networks through residual blocks that enable stable training by learning residual mappings via shortcut connections. Recent adaptations of ResNet variants for prostate MRI segmentation have further optimized this backbone: Gurkan et al. (16) integrated ResNet50 as the encoder within a Mask R-CNN framework, leveraging its feature reuse capabilities to improve segmentation of anatomically distinct prostate zones by aligning region proposals with high-resolution feature maps. Similarly, Li et al. (17) enhanced ResNet50's context capture by replacing standard convolutions with dilated convolutions in deeper layers, strategically expanding the network's receptive fields without increasing computational overhead a critical advantage for resolving subtle intensity variations in T2-weighted MRI. Talaat et al. (18) integrates ResNet50 with Faster R-CNN and dual optimizers, aiming to improve prostate cancer detection accuracy. This model demonstrates significant performance improvements in detecting and localizing prostate lesions in MRI images. Another study focuses on utilizing ResNet50 for feature extraction, achieving high classification accuracy in detecting prostate cancer lesions, and providing a robust solution for automated cancer detection (19). Furthermore, the MM-UNet architecture combines a modified ResNet50 encoder with Mamba blocks, refining feature extraction and enhancing segmentation precision in prostate MRI scans. This model not only improves segmentation accuracy but also aids in resolving complex boundaries within the prostate zone (20). These targeted modifications demonstrate how ResNet50's residual learning principles can be tailored to balance model depth and efficiency while preserving the precision required for prostate zonal anatomy segmentation.

### 2.2 Attention mechanisms in skip connections

Attention gates refine the propagation of features between the encoder and decoder by adaptively suppressing irrelevant regions in skip connections. The seminal Attention U-Net (21) introduced channel-wise attention mechanisms, where feature maps from the encoder are weighted using attention coefficients derived from the decoder's higher-level features, enabling the model to focus on salient regions like pancreatic tumors while ignoring background noise. For prostate MRI segmentation, Nash et al. (22) extended the use of attention mechanisms by incorporating spatial attention into skip connections. This approach generates pixel-wise attention maps that focus on anatomically plausible boundaries of the prostate, leveraging learned spatial correlations between the encoder and decoder features. Building on this, Zhang et al. (23) further improved robustness against MRI intensity inhomogeneity by integrating self-attention mechanisms with deformable convolutions in the skip connections. The self-attention captures long-range dependencies to resolve ambiguous edges, while deformable convolutions adjust the receptive fields to better accommodate irregular prostate shapes. Federico et al. (24) proposes Long-Range 3D Self-Attention to capture multi-scale features in MRI scans, improving segmentation accuracy. Another introduces a pseudo-3D Global–Local Channel Spatial Attention mechanism to enhance segmentation of prostate zones in T2-weighted MRI, significantly improving accuracy for both the transition and peripheral zones (25). Building on these advancements, our hybrid channel-spatial attention modules unify channel-wise and spatial attention within skip connections, dynamically amplifying discriminative prostate features (e.g., subtle intensity gradients) while suppressing confounding background signals through joint optimization of channel relevance and spatial saliency.

### 2.3 Multi-scale feature fusion

Effective fusion of hierarchical features is critical for segmenting small, ambiguous structures like the prostate, where local texture details and global anatomical context must be cohesively integrated. Zhao et al. (26) introduced Feature Pyramid Networks (FPNs), which merge multi-scale encoder outputs through lateral connections, creating a pyramid of features that combines high-resolution shallow layers (rich in spatial details) with semantically strong deeper layers (capturing contextual information). For prostate MRI segmentation, Santhirasekaram et al. (27) adapted this approach by incorporating geometric constraints into the fusion process, penalizing segmentations that violate anatomical priors (e.g., irregular prostate topology) through a loss term that enforces smoothness and connectivity in the fused feature maps. Li et al. (28) further advanced multi-scale fusion by introducing learnable weights to dynamically adjust the contribution of ResNet50's intermediate features during aggregation, enabling the model to prioritize scales resilient to common MRI artifacts such as motion blur or intensity inhomogeneity emphasize multi-scale feature fusion for prostate segmentation in MRI. AGMSF-Net integrates a multi-scale attention mechanism and 3D transformer module, improving segmentation accuracy and achieving a DSC of 93.68% on a local dataset (29). Another approach uses a multistream fusion encoder with spatial attention maps, enhancing accuracy, particularly for small lesions, and achieving improved performance on the ProstateX dataset (30).

## 3 Methodology

**Figure 1:**
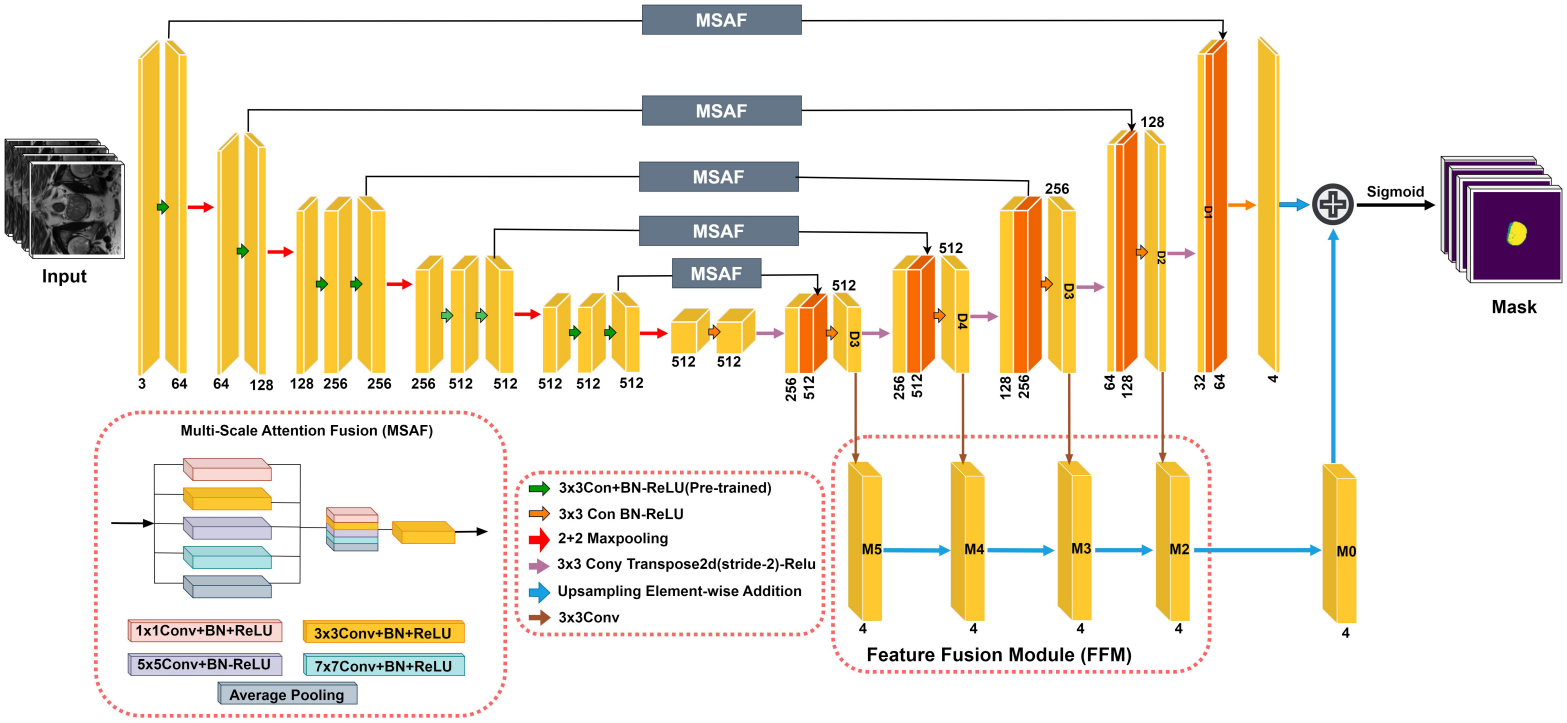
Proposed RaNet framework with MSAF and FFM on left and right respectively.

The RaNet model (Figure 1), designed for medical image segmentation tasks, follows an encoder-decoder architecture enhanced with attention mechanisms and feature fusion techniques. The encoder consists of convolutional blocks with residual connections to preserve spatial integrity while extracting high-level features. The attention mechanism, implemented through the MSAF, refines feature maps by focusing on relevant regions using varying kernel sizes and dilation rates, improving segmentation accuracy. The decoder mirrors the encoder with deconvolutional layers and skip connections to retain essential spatial details for accurate boundary delineation. Additionally, the Feature Fusion Module (FFM) merges outputs from multiple decoder layers using bilinear upsampling, ensuring robust and precise segmentation. The final segmentation mask is computed by combining these refined features, supporting accurate diagnosis and treatment planning. This architecture optimizes feature retention and relevance, significantly enhancing segmentation performance in medical imaging. Overall component summary of the RaNet is given in the Table 1.

**Table 1:** Summary of model components.

**Component**	**Description**
DCNet	Modified ResNet50 with dilated convolutions and removed initial max-pooling.
MSAF	Multi-scale attention fusion with varying kernel sizes and dilation rates.
FFM	Bilinear upsampling to merge decoder outputs, enhancing segmentation accuracy.
Fusion strategy	Combines outputs from multiple layers to retain both spatial and semantic info.
Loss function	Hybrid loss combining Dice, weighted cross-entropy, and boundary loss.

### 3.1 DilatedContextNet

In this paper, we enhance the ResNet50 architecture by making three key modifications to the encoder (the feature extraction part) of the network. These modifications aim to preserve spatial information, improve feature extraction for segmentation tasks, and introduce uncertainty modeling. In the original ResNet50, a max-pooling layer is applied immediately after the first convolutional layer. However, max-pooling reduces the spatial resolution of the feature maps, which is undesirable for segmentation tasks where fine-grained spatial details are critical. To address this issue, we remove the initial max-pooling layer, preserving the spatial dimensions of the input image as it passes through the network. Let the input to the encoder be represented as:


(1) $$\begin{array}{l} X_{0}\in {\mathbb{R}}^{H_{0}\times W_{0}\times C_{0}} \end{array}$$

where *H*_0_, *W*_0_, and *C*_0_ represent the height, width, and number of channels of the input image. In the original ResNet50, the max-pooling operation would reduce the spatial dimensions as:


(2) $$\begin{array}{l} X_{1}=\text{MaxPool}\left(X_{0}\right) \end{array}$$

However, in the modified version, we remove this step and directly pass *X*_0_ through the first convolutional layer:


(3) $$\begin{array}{l} X_{1}=\text{Conv}\left(X_{0}\right) \end{array}$$

Thus, we avoid any early downsampling, allowing the network to retain the original spatial resolution.

The bottleneck block in the fourth layer has a stride of 2, which reduces the spatial resolution of the feature maps too aggressively for segmentation. To address this, we replace the stride-2 bottleneck block with a regular convolutional block with stride 1. This ensures that the spatial resolution is maintained in the fourth layer, allowing the network to retain more detailed features.

The regular convolution operation is mathematically represented as:


(4) $$\begin{array}{l} X_{2}=\text{Conv}\left(X_{1},\text{stride}=1\right) \end{array}$$

where *X*_1_ is the output from the previous layer and the stride is set to 1 to preserve spatial dimensions.

To further improve the ability of the network to capture larger contextual information while maintaining spatial resolution, we introduce a dilated convolution in the second block of the fourth layer. Dilated convolutions increase the receptive field by introducing gaps between the convolutional kernel's elements, allowing the network to capture larger contextual information without downsampling the feature maps.

The dilated convolution operation with dilation rate *r* is expressed as:


(5) $$\begin{array}{l} \text{DilatedConv}\left(X,r\right)=\overset{k}{\underset{i=1}{\sum }}w_{i}X_{i\cdot r} \end{array}$$

where *w*_*i*_ represents the convolutional kernel, *X*_*i*·*r*_ represents the dilated input, and *r* is the dilation rate. By using a dilated convolution in the second block of the fourth layer, we prevent spatial information loss while capturing a broader context.

Thus, the output from the dilated bottleneck block is:


(6) $$\begin{array}{l} X_{3}=\text{DilatedConv}\left(X_{2},r\right) \end{array}$$

where *X*_2_ is the output from the previous regular convolutional block.

To transform the DCNet into a Bayesian neural network, we incorporate dropout layers after each block. Dropout is a regularization technique that randomly deactivates a proportion of neurons during training, helping the model generalize better by preventing overfitting. In a Bayesian context, dropout can be interpreted as a way to approximate the posterior distribution of the network's weights, making the network more robust and capable of modeling uncertainty.

Mathematically, the output of a neuron with dropout is given by:


(7) $${\hat{y}}_{i}=\{\begin{array}{ll} y_{i} & \text{with probability}p\\ 0 & \text{with probability}(1-p) \end{array}$$

where *p* is the probability of keeping the neuron active (typically *p* = 0.5).

For each block in the encoder, dropout is applied as follows:


(8) $$\begin{array}{l} X_{i}^{\prime }=\text{Dropout}\left(X_{i},p\right) \end{array}$$

where *X*_*i*_ represents the output from the *i*-th block, and *p* is the dropout rate.

This DCNet architecture aim to enhance prostate segmentation, crucial for prostate cancer diagnosis. Removing the initial max-pooling layer preserves fine spatial details essential for accurate boundary detection. Replacing the stride-2 bottleneck with a regular convolution and introducing dilated convolutions retain spatial resolution while expanding the receptive field to capture broader tissue context. Incorporating dropout layers converts the model into a Bayesian neural network, improving generalization and robustness.

### 3.2 Multi-scale attention fusion

The architecture of the MSAF is depicted in Figure 1. During the feature map learning phase from the encoder, convolution operations are performed simultaneously before being directly connected to the decoder. The resulting feature maps are then concatenated. To reduce the number of channels, a transition block consisting of a convolution layer followed by a ReLU activation function (Conv + PreLU) is added. Various methods for concatenating convolutional layers with different kernel sizes and dilation rates were explored. The optimal MSAF architecture, chosen from experimental comparisons and shown in Figure 1, incorporates a 1 × 1 Conv + PreLU block, a 3 × 3 Conv + PreLU block with dilation rate 1, a 3 × 3 Conv + PreLU block with dilation rate 2, a 3 × 3 Conv + PreLU block with dilation rate 3, and an image pooling layer. The overall procedure of MSAF can be mathematically expressed as:


(9) $$\begin{array}{l} X_{N}=f(\text{Concat}(K_{1}(X^{O}),K_{3}(X^{O},\text{D}=1),K_{3}(X^{O},\text{D}=2),\\ \;K_{3}(X^{O},\text{D}=3),G_{I}(X^{O}))) \end{array}$$

where *X*_*O*_ represents the features from the encoder, *X*_*N*_ denotes the new features generated by MSAF, which are then passed to the decoder. *K*_*i*_ refers to the convolution operations with kernel size *i*, and *GI* stands for the global image pooling operation. The function *f*(·) is the transition operation that adjusts the number of channels in *X*_*N*_ to match that of *X*_*O*_.

The MSAF structure bears similarities to the X-ception module, which provides several advantages, such as the ability to capture multi-scale information through different convolution kernel sizes. This is particularly useful for real-world applications where target segmentation requires handling scale variance. Additionally, as demonstrated in GoogleNet, the use of multiple kernel sizes can facilitate faster convergence by decomposing sparse matrices into dense matrix operations. To further enhance the model's performance, a global image average pooling operation is incorporated, which has been shown to effectively capture global context information in several studies.

### 3.3 Feature fusion module

In U-Net-based models, probability maps are generated by the final layer of the decoder. Since feature maps in convolutional networks cannot simultaneously retain both semantic and spatial information, robust and accurate probability maps are obtained by fusing the outputs from different decoder layers. This process can be formulated as follows:


(10) $$\begin{array}{l} F_{o}=\overset{5}{\underset{i=2}{\sum }}U_{u}\left(F_{i}\right) \end{array}$$

where *F*_*o*_ represents the fused feature map generated by the FFM, and *F*_*i*_ corresponds to the feature map produced by decoder layer *D*_*i*_ (for *i* = 2, 3, 4, 5). The function *U*_*u*_ denotes bilinear upsampling, which is used to adjust the probability map size to match the original image dimensions.

The final segmentation mask is computed as:


(11) $$\begin{array}{l} \text{Mask}=\text{Sigmoid}\left(F_{1}+F_{o}\right) \end{array}$$

where *F*_1_ is the feature map from the last decoder layer *D*_1_, and the segmentation mask is obtained by applying the sigmoid function to the sum of *F*_1_ and *F*_*o*_.

### 3.4 Loss function

To address the challenges of segmenting the prostate in MRI, which exhibits ambiguous boundaries and class imbalance between foreground and background, we design a hybrid loss function. The total loss $L_{\text{total}}$ combines three components: a region-based Dice loss ($L_{\text{Dice}}$), a distribution-aware weighted cross-entropy loss ($L_{\text{WCE}}$), and a boundary-focused loss ($L_{\text{boundary}}$) to refine edge delineation. The combined loss is defined as:


(12) $$\begin{array}{l} L_{\text{total}}=\lambda_{1}L_{\text{Dice}}+\lambda_{2}L_{\text{WCE}}+\lambda_{3}L_{\text{boundary}}, \end{array}$$

where λ_1_, λ_2_, and λ_3_ are weighting coefficients balancing the contributions of each term.

**Dice loss** **(${ℒ}_{\text{Dice}}$)** The Dice loss (31) mitigates class imbalance by maximizing the overlap between the predicted segmentation mask ŷ and ground truth *y*:


(13) $${ℒ}_{\text{Dice}}=1-\frac{2\sum_{i}y_{i}{\hat{y}}_{i}+\epsilon }{\sum_{i}y_{i}+\sum_{i}{\hat{y}}_{i}+\epsilon }$$

where ϵ is a smoothing factor to avoid division by zero.

**Weighted cross-entropy loss** **(${ℒ}_{\text{WCE}}$)** To penalize misclassifications in underrepresented prostate regions, we use a weighted cross-entropy loss (4):


(14) $${ℒ}_{\text{WCE}}=-\sum_{i}w\cdot \left[y_{i}\log({\hat{y}}_{i})+(1-y_{i})\log(1-{\hat{y}}_{i})\right]$$

where *w* is a class weight inversely proportional to the foreground pixel frequency.

**Boundary loss** **(${ℒ}_{\text{boundary}}$)** To improve segmentation accuracy along poorly defined prostate edges, we adopt a boundary loss (32) that computes the symmetric Euclidean distance between the contours of *y* and ŷ:


(15) $$\begin{array}{l} L_{\text{boundary}}=\underset{p\in \partial y}{\sum }\underset{q\in \partial ŷ}{\min}||p-q|{|}_{2}+\underset{q\in \partial ŷ}{\sum }\underset{p\in \partial y}{\min}||q-p|{|}_{2} \end{array}$$

where ∂*y* and ∂ŷ denote the contours of the ground truth and prediction, respectively.

## 4 Dataset and pre-processing

### 4.1 Dataset

#### 4.1.1 PROMISE12

**Figure 2:**
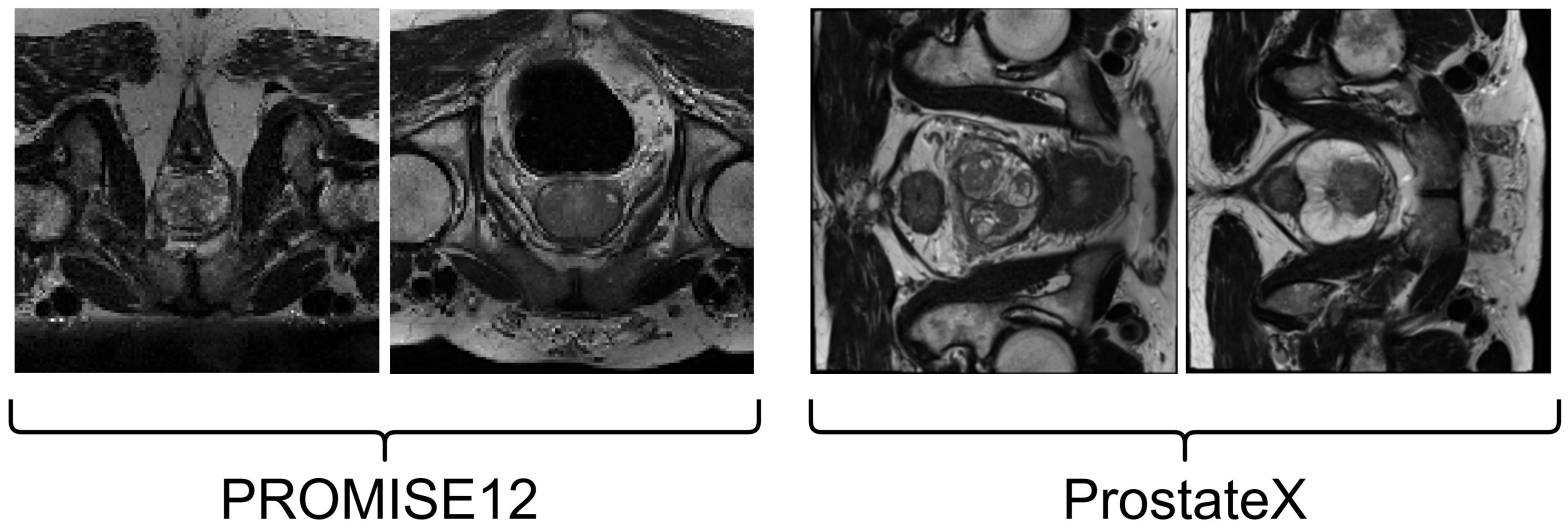
Image samples from PROMISE12 dataset **(on Left)**, ProstateX dataset **(on Right)**.

The prostate MRI data used in this study are sourced from the PROMISE12 Challenge dataset (33). The PROMISE12 Challenge dataset is designed for studying MRI prostate segmentation and comprises 50 T2 MRI scans of the prostate region from 50 patients. The data were collected from multiple hospitals, ensuring representation of a clinical setting with diverse vendors and acquisition protocols. Specifically, the dataset includes 50 MRI volumes with corresponding training labels and 30 MRI volumes for testing, which lack ground truth images. A few image samples are shown in Figure 2.

#### 4.1.2 ProstateX

The ProstateX dataset, originally part of the PI-CAI dataset, did not include segmentation masks for anatomical regions. However, a subsequent study by Cuocolo et al. (34) annotated 204 cases from the original ProstateX dataset, providing both lesion masks and anatomical region masks. This enhanced dataset is valuable for research in lesion detection and anatomical region segmentation in prostate imaging. Example images from this dataset are shown in Figure 2.

### 4.2 Evaluation metrics

The performance of the proposed RaNet model for segmenting the prostate zones in T2-weighted MRI is evaluated using the Dice coefficient, Intersection over Union (IoU), and accuracy. The Dice coefficient measures the similarity between the predicted segmentation mask of the prostate zones and the ground truth mask, while IoU assesses the overlap between these masks. Accuracy represents the proportion of correctly predicted pixels in the segmentation task.


(16) $$\begin{array}{l} \text{DSC}=\frac{2\cdot \text{intersection}\left(A,B\right)}{\text{size}\left(A\right)+\text{size}\left(B\right)} \end{array}$$

(17) $$\begin{array}{l} \text{IoU}=\frac{\text{intersection}\left(A,B\right)}{\text{union}\left(A,B\right)} \end{array}$$

(18) $$\begin{array}{l} \text{Accuracy}=\frac{\text{intersection}\left(A,B\right)}{\text{union}\left(A,B\right)} \end{array}$$

The Dice Similarity Coefficient (DSC) quantifies the overlap between the predicted prostate zone segmentation mask and the ground truth mask *A* and *B*, considering both false positives and false negatives. The Intersection Over Union (IoU) measures the proportion of overlap between the predicted and ground-truth masks, normalized by their union. Accuracy measures the fraction of correctly identified pixels in the segmentation task by comparing the intersection of the sets with their union.

### 4.3 Implementation details

The experiments were conducted on a single NVIDIA RTX 3090 GPU using the PyTorch framework. The input images were resized to 256 × 256 pixels, and pixel intensities and voxel resolutions were standardized to enhance model generalizability. The data was split into 70% for training, 20% for validation, and 10% for testing. Both datasets underwent comprehensive preprocessing, which included a structured hierarchy of techniques, such as various augmentation strategies (no augmentation, vertical, horizontal, and diagonal shifts) and a mix of original and downsampled data. To optimize model training, hyperparameters were fine-tuned by exploring batch sizes (4, 8, and 16) and learning rates (1 × 10^−2^, 1 × 10^−3^, and 1 × 10^−4^). Each combination was evaluated using metrics like accuracy, precision, specificity, DSC and Jaccard to assess its effectiveness. The augmentation and preprocessing parameters are given in Table 2. Additionally, preprocessing metrics for the PROMISE12 dataset are provided in Table 3, while those for the ProstateX dataset are outlined in Table 4. The model was trained end-to-end using the Adam optimizer (35). A ReduceLROnPlateau learning rate scheduler dynamically adjusted the learning rate during training. The model was trained for a maximum of 200 epochs with early stopping based on validation loss. To address the challenges of prostate segmentation, a hybrid loss function was employed to manage class imbalance and improve edge delineation.

**Table 2:** Augmentation and preprocessing parameters for the experiment.

**Attributes**	**Description**
Horizontal shift	True
Zoom	True
Vertical shift	True
Diagonal shift	True
Hue saturation	True
Random brightness	True
Random contrast	True

**Table 3:** Performance evaluation with different pre-processing and hyper-parameter settings on PROMISE12.

			**Accuracy**	**Precision**	**Specificity**	**DSC**	**Jaccard**

Pre-processing	Data augment	None	95.81	94.61	94.23	92.57	87.92
				Vertical	96.54	95.12	95.58	93.39	88.71
				Horizontal	96.89	95.49	96.14	95.55	89.19
				Diagonal	98.21	95.88	95.95	97.66	89.96
Hyper-parameters	Batch	4	97.83	93.79	94.15	92.21	86.65
				8	98.19	94.42	97.91	92.73	87.37
				16	98.78	95.44	95.71	93.77	88.25
		Learning rate	1 × 10^−2^	97.63	91.32	95.75	95.82	86.47
				1 × 10^−3^	99.61	92.76	97.91	97.54	87.83
				1 × 10^−4^	98.51	92.19	94.23	94.62	86.84

**Table 4:** Performance evaluation with different pre-processing and hyper-parameter settings on ProstateX.

			**Accuracy**	**Precision**	**Specificity**	**DSC**	**Jaccard**

Pre-processing	Data augment	None	94.82	90.38	89.13	90.28	83.29
				Vertical	96.43	91.61	92.72	91.94	85.97
				Horizontal	95.37	91.17	92.20	91.32	84.23
				Diagonal	97.71	92.52	93.89	92.65	86.48
Hyper-parameters	Batch	4	95.46	89.91	89.37	90.13	85.28
				8	92.73	91.57	91.53	91.42	86.65
				16	96.51	91.67	91.19	92.76	87.88
		Learning Rate	1 × 10^−2^	93.85	91.24	91.49	90.71	84.72
				1 × 10^−3^	97.44	90.62	93.37	93.17	86.58
				1 × 10^−4^	94.14	92.29	92.86	91.34	86.73

## 5 Results and analysis

### 5.1 Comparison with different encoders

The comparison of different encoders with our proposed modules on the PROMISE12 dataset shows significant changes in performance across various models (Table 5). Starting with ResNet-18, the baseline model, we observe an improvement with ResNet-34, which shows a DSC increase of 1.67%, IoU improvement of 1.65%, and an accuracy boost of 2.5%. These improvements are attributed to ResNet-34's deeper architecture, which allows for better feature extraction through residual connections. Further improvements are seen with ResNet-50, which achieves a DSC increase of 0.88%, IoU improvement of 0.24%, and a slight decrease in accuracy of 0.25%. This increase in DSC and IoU can be attributed to the enhanced feature extraction capabilities and deeper layers of ResNet-50, allowing it to capture more detailed spatial features. The EfficientNet models show more varied results. EfficientNetB3 has a slight decrease in DSC (down by 0.75%) and a small increase in specificity (up by 0.43%) compared to ResNet-50, but its accuracy and IoU are lower than ResNet-50. This suggests that while EfficientNetB3 is more efficient, it may not be able to capture the same level of detail as deeper models like ResNet-50. EfficientNetB4, on the other hand, achieves a DSC increase of 0.44%, IoU improvement of 0.36%, and accuracy increase of 0.57%, indicating that a deeper and more optimized EfficientNet performs well while maintaining computational efficiency. EfficientNetB5 shows a small improvement in DSC (up by 0.20%) and IoU (up by 0.12%), while its accuracy increases by 0.54% compared to ResNet-50. Finally, RaNet outperforms all models, with a DSC increase of 1.69% over EfficientNetB5, an IoU improvement of 0.43%, and a significant accuracy gain of 1.30%. This improvement is due to RaNet's use of dilated convolutions, attention mechanisms, and the removal of max-pooling layers, which enhance feature retention, preserve spatial resolution, and refine segmentation performance.

**Table 5:** Comparison with different encoders on PROMISE12.

**Model**	**DSC%**	**Sensitivity%**	**Specificity%**	**Accuracy%**	**IoU%**
ResNet-18	94.37	86.71	93.46	96.07	95.47
ResNet-34	96.04	88.89	95.38	98.57	97.12
ResNet-50	96.92	87.16	96.10	98.32	97.36
EfficientNetB3	96.17	86.94	96.29	97.15	95.84
EfficientNetB4	95.61	87.25	97.54	97.89	96.72
EfficientNetB5	96.32	88.39	96.67	98.43	97.26
RaNet	98.61	89.42	98.90	99.73	97.69

On the ProstateX dataset (Table 6), RaNet also demonstrates notable improvements and variations in performance. Starting with ResNet-18, the baseline model, we observe a DSC improvement of 2.75%, IoU increase of 2.59%, and accuracy gain of 1.75% with ResNet-34. This improvement is due to ResNet-34's deeper architecture, which allows for better feature extraction and handling of more complex patterns through residual connections. Further increases are observed with ResNet-50, which shows a DSC increase of 0.68%, IoU improvement of 0.74%, and accuracy improvement of 0.09% compared to ResNet-34. The additional layers in ResNet-50 refine feature extraction and improve segmentation, capturing more detailed and complex spatial information. In contrast, the EfficientNet models show varying performance. EfficientNetB3 underperforms compared to ResNet-50, with a DSC decrease of 2.09% and IoU decrease of 4.05%. This decrement is likely due to EfficientNetB3's compact design, which prioritizes computational efficiency but may not capture as detailed features as deeper models like ResNet-50. EfficientNetB4, however, shows a slight improvement over ResNet-50, with a DSC increase of 0.68%, IoU increase of 0.74%, and accuracy increase of 0.54%. This indicates that a deeper and more optimized EfficientNet model benefits from better feature extraction while maintaining efficiency. EfficientNetB5 shows a small decrement in DSC (down by 1.52%) and IoU (down by 0.79%), likely due to diminishing returns as model depth increases without corresponding performance gains.

**Table 6:** Comparison with different encoders on ProstateX.

**Model**	**DSC%**	**Sensitivity%**	**Specificity%**	**Accuracy%**	**IoU%**
ResNet-18	92.19	84.63	92.17	96.56	92.30
ResNet-34	94.94	86.72	94.80	98.31	94.89
ResNet-50	95.62	87.43	96.35	98.40	95.42
EfficientNetB3	93.53	84.59	93.16	94.38	91.37
EfficientNetB4	95.29	86.41	96.28	96.94	94.56
EfficientNetB5	94.10	86.68	95.83	96.61	94.63
RaNet	96.57	87.49	96.74	97.26	95.16

Finally, RaNet outperforms all models, achieving a DSC increase of 0.95%, IoU improvement of 0.74%, and an accuracy gain of 0.86%. This can be attributed to RaNet's architecture, which utilizes dilated convolutions, removes max-pooling layers, and integrates attention mechanisms, resulting in superior feature retention and refined segmentation performance.

### 5.2 Ablation study

The ablation study demonstrates the performance improvement with each module added to the base UNet architecture (Table 7). Starting with the base UNet model, it provides solid performance, achieving a DSC of 94.82% and accuracy of 95.52% on PROMISE12, and a DSC of 93.16% and accuracy of 93.17% on ProstateX. The first improvement comes with the addition of DCNet, which modifies the ResNet50 backbone by removing the initial max-pooling layer and replacing the stride-2 bottleneck with a regular convolution. These modifications preserve spatial resolution and enhance the model's ability to capture finer details. The inclusion of DCNet results in an increase in DSC of 1.49% and an improvement in precision of 0.91% on PROMISE12, while on ProstateX, the DSC increases by 1.70% and the accuracy by 2.49%. Adding the MSAF module introduces multi-scale feature fusion, allowing the model to capture features at varying scales. This module improves the model's ability to focus on relevant regions with greater precision, leading to improved segmentation of complex structures like the prostate. MSAF results in an increase in DSC of 1.35% and an improvement in accuracy of 2.46% in PROMISE12, and in ProstateX, the DSC increases by 0.93% and the accuracy by 1.07%. The final module, FFM merges the outputs from multiple decoder layers using bilinear upsampling, ensuring that both spatial and semantic information are preserved. This module strengthens the segmentation by consolidating the features learned at different levels of the network. The addition of FFM leads to a DSC increase of 0.95% and an accuracy boost of 0.84% on PROMISE12, and on ProstateX, DSC increases by 0.78% and accuracy by 0.53%. In conclusion, the integration of DCNet, MSAF, and FFM results in significant performance improvements, with RaNet achieving a DSC of 98.61% and accuracy of 99.73% on PROMISE12, and a DSC of 96.57% and accuracy of 97.26% on ProstateX, demonstrating the effectiveness of the combined architecture for prostate zone segmentation.

**Table 7:** Ablation performance comparison between PROMISE12 and ProstateX.

	**PROMISE12**		**ProstateX**	

**Models**	**DSC%**	**Accuracy%**	**DSC%**	**Accuracy%**
UNet	94.82	95.52	93.16	93.17
UNet + DCNet	96.31	96.43	94.86	95.66
UNet + DCNet + MSAF	97.66	98.89	95.79	96.73
UNet + DCNet + MSAF + FFM	98.61	99.73	96.57	97.26

### 5.3 Comparison with state-of-the-art methods

The performance comparison of RaNet with state-of-the-art prostate segmentation models on the PROMISE12 dataset (Table 8) reveals significant improvements. Compared to the original UNet (4), RaNet achieves a DSC increase of 3.79%, an IoU improvement of 4.93%, and an accuracy boost of 4.21%. These gains are attributed to RaNet's use of dilated convolutions, attention mechanisms, and the removal of max-pooling layers, which help preserve spatial resolution and refine feature maps. When compared to MicroSeg-Net (10), which uses multi-scale feature fusion and attention mechanisms, RaNet shows a DSC increase of 3.09%, IoU improvement of 3.12%, and accuracy increase of 2.60%, highlighting its superior feature retention and spatial resolution. Against PZS-Net (36), which incorporates a pyramid structure and attention layers, RaNet demonstrates a DSC increase of 2.32% and IoU improvement of 1.64%, thanks to its more effective attention-based fusion and feature extraction capabilities. Finally, RaNet outperforms nnUNet (37), which is known for its adaptive design and strong performance, by showing a DSC improvement of 1.66%, an IoU increase of 1.21%, and an accuracy boost of 1.13%. This performance is due to RaNet's ability to capture both fine details and larger contextual information through its dilated convolutions and attention mechanisms. Overall, RaNet outperforms all SOTA models, with significant improvements in DSC, IoU, and accuracy, demonstrating the effectiveness of its architecture in prostate zone segmentation. Comparison with other previous work for PROMISE12 dataset is given in Table 9.

**Table 8:** State-of-the-art comparison on PROMISE12.

**Model**	**DSC%**	**Sensitivity%**	**Specificity%**	**Accuracy%**	**IoU%**
UNet (4)	94.82	84.23	91.11	95.52	92.76
MicroSeg-Net (10)	95.52	87.83	94.27	97.13	94.57
PZS-Net (36)	96.29	88.91	96.89	98.99	96.05
nnUNet (37)	96.95	87.97	97.11	98.60	96.48
RaNet	98.61	89.42	98.90	99.73	97.69

**Table 9:** Comparison with previous methods on Promise12.

**References**	**Technique**	**Dataset**	**Cases**	**DSC%**
Jia et al. (38)	3D APA-Net	PROMISE12, ASPS13	140	90.10
Zhu et al. (39)	BOWDA-Net	PROMISE12, BWH	146	92.54
Wang et al. (40)	SegDGAN	PROMISE12, Decathlon, ISBI13, QIN-PROSTATE	335	91.66
Qian et al. (41)	ProSegNet	PROMISE12, ProstateX	80	90.80
Meyer et al. (42)	Multi-Stream-CNN	PROMISE12, In-house dataset, ProstateX	19	93.00
Ocal et al. (43)	Triple Fusion Model	PROMISE12, NCI-ISBI 2013	80	91.90
Jia et al. (44)	MSD-Net	PROMISE12, 12CVB, NCI-ISBI13	180	92.90
Chen et al. (45)	RASEU-Net	PROMISE12, Private Dataset	30	80.70
Li et al. (28)	DRCU-Net	PROMISE12	80	91.60
Bhandary et al. (46)	nnU-Net	PROMISE12, Medical Segmentation Decathlon	50	91.20
Ma et al. (47)	ResGNet	PROMISE12, Prostate158, NCI-ISBI13, PI-CAI	1,764	94.40
Ours	RaNet	PROMISE12	50	98.61

Table 10 presents the performance comparison of RaNet with state-of-the-art prostate segmentation models on the ProstateX dataset, showing clear improvements. Compared to the original UNet (4), RaNet achieves a DSC increase of 3.41%, an IoU improvement of 4.90%, and an accuracy boost of 4.09%. These significant improvements stem from RaNet's architectural innovations, such as dilated convolutions, attention mechanisms, and the removal of max-pooling layers, which preserve spatial resolution and refine feature maps. When compared to MicroSeg-Net (10), which uses multi-scale feature fusion and attention mechanisms, RaNet shows a DSC increase of 2.65%, IoU improvement of 3.30%, and accuracy increase of 2.70%, highlighting RaNet's superior spatial resolution and attention-based fusion strategy. In comparison to PZS-Net (36), which incorporates pyramid structures and attention layers, RaNet demonstrates a DSC increase of 1.41% and an IoU improvement of 1.73%, benefiting from its more refined attention-based fusion and feature retention capabilities. Finally, RaNet outperforms nnUNet (37), achieving a DSC improvement of 0.75%, IoU increase of 1.09%, and accuracy boost of 0.89%. The performance gains of RaNet are attributed to its advanced architecture, which captures both fine details and larger contextual information through dilated convolutions and attention mechanisms. Overall, RaNet demonstrates superior performance across all metrics, with substantial improvements over all other SOTA models, highlighting the effectiveness of its architecture for prostate zone segmentation on the ProstateX dataset. Comparison with other previous work for ProstateX dataset is given in Table 11.

**Table 10:** State-of-the-art comparison on ProstateX.

**Model**	**DSC%**	**Sensitivity%**	**Specificity%**	**Accuracy%**	**IoU%**
UNet (4)	93.16	82.19	90.14	93.17	90.26
MicroSeg-Net (10)	93.92	85.42	94.27	94.56	91.86
PZS-Net (36)	95.16	85.96	95.54	95.91	93.43
nnUNet (37)	95.82	86.64	96.18	96.37	94.07
RaNet	96.57	87.49	96.74	97.26	95.16

**Table 11:** Comparison with previous methods on ProstateX.

**References**	**Technique**	**Dataset**	**Cases**	**DSC%**
Yu et al. (48)	SPCT	ProstateX	914	92.23
Zhong et al. (49)	ProSegDiff	ProstateX, NCI-ISBI, PROMISE12	-	89.16
Qian et al. (41)	ProSegNet	ProstateX, PROMISE12	346	89.20
Liu et al. (50)	CriDiff	ProstateX, NCI-ISBI	-	87.40
Yan et al. (51)	CCT-Unet	ProstateX, Huashan dataset	200	80.30
Hung et al. (52)	CAT-nnU-Net	ProstateX, private dataset	193	83.90
Nguyen and Fernandez-Quilez (53)	nnU-Net	ProstateX	204	98.00
Wei et al. (54)	attention U-Net	ProstateX, Prostate158, MSD	443	82.00
Nai et al. (55)	HighRes3DNet	ProstateX	160	89.00
Ours	RaNet	ProstateX	204	96.57

**Figure 3:**
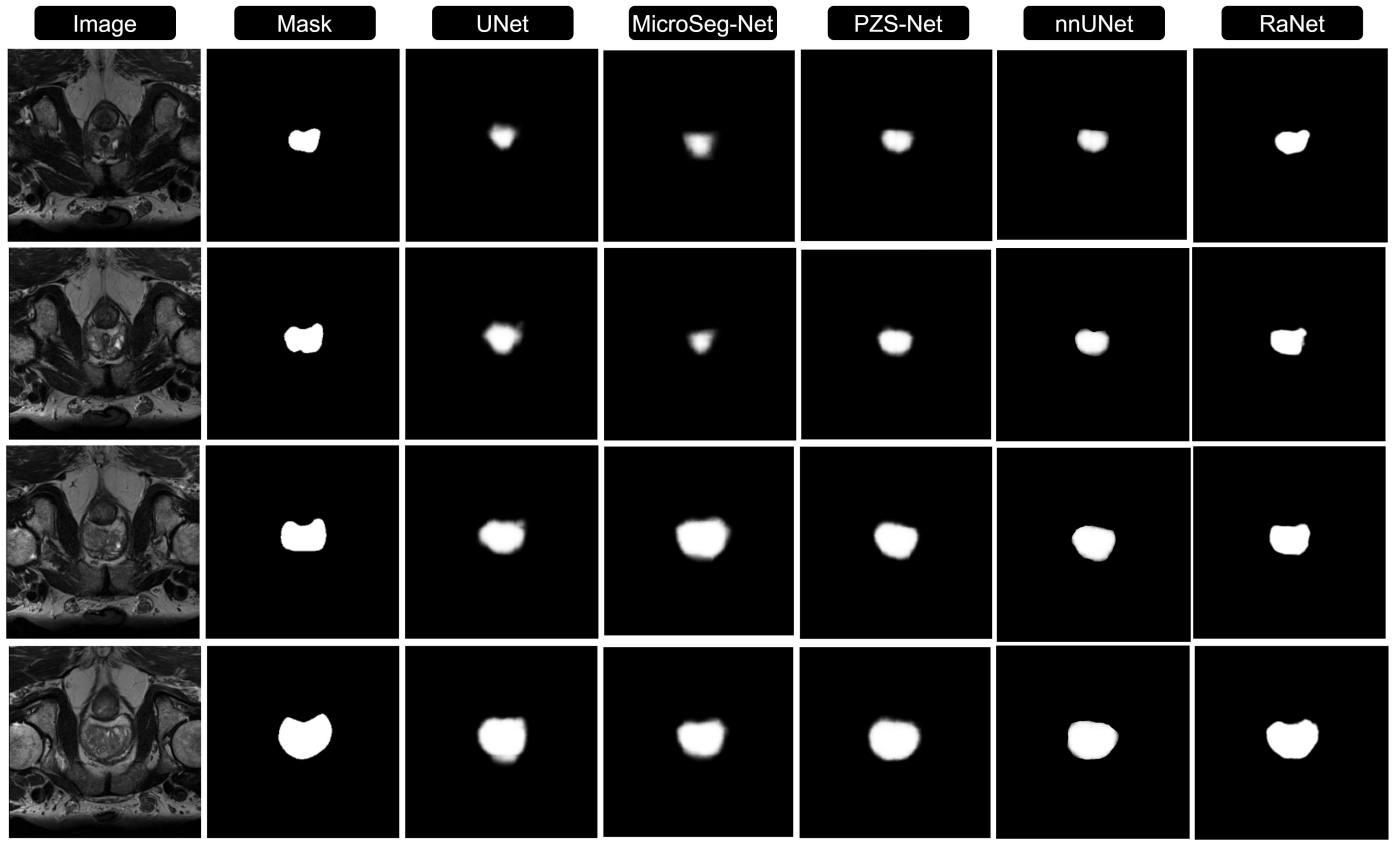
Comparison of RaNet's segmentation performance with different state-of-the-art methods on PROMISE12 dataset. From left to right: input image, ground truth, UNet, MicroSeg-Net, PZS-Net, nnUNet, and RaNet.

**Figure 4:**
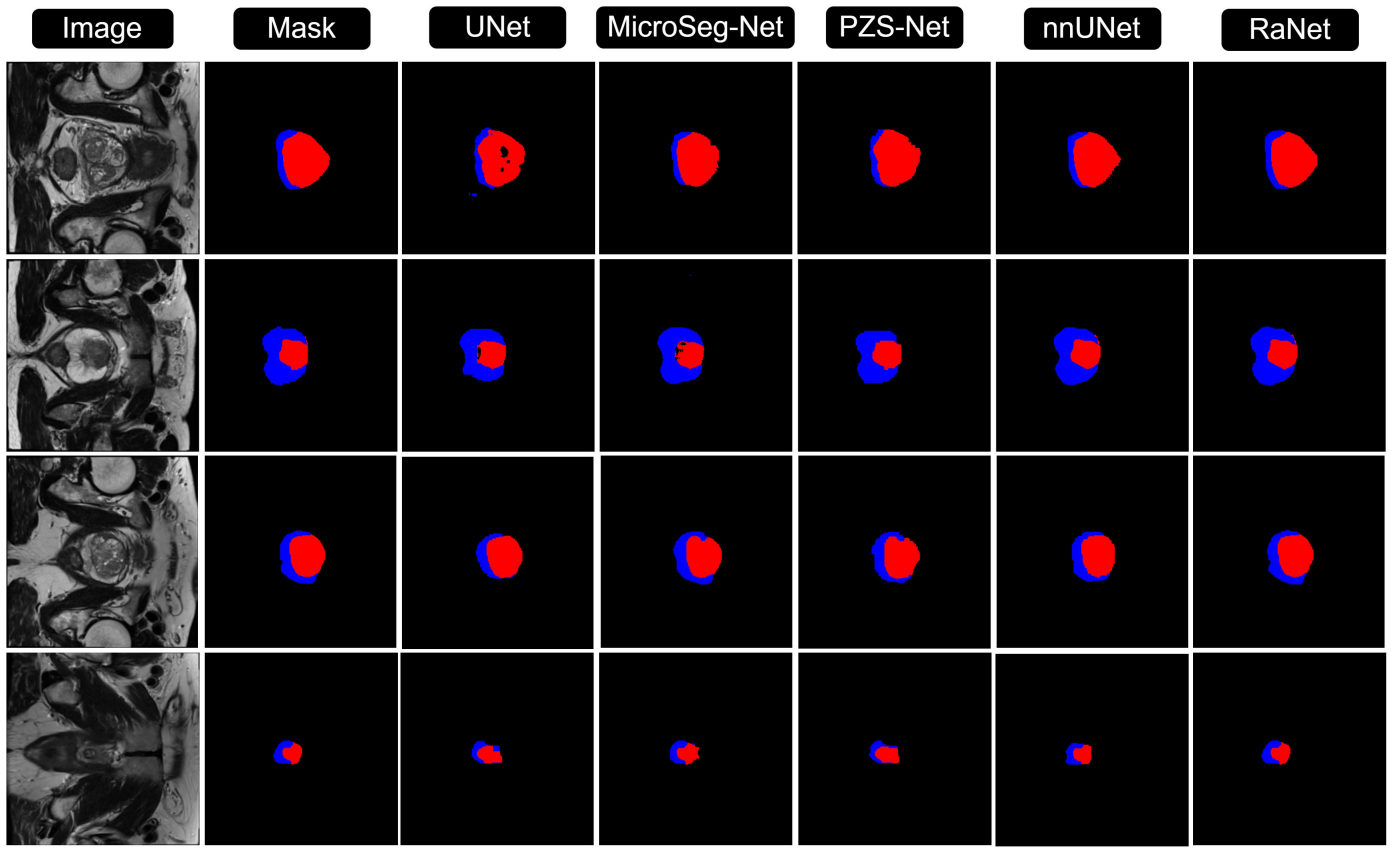
Comparison of RaNet's segmentation performance with different state-of-the-art methods on ProstateX dataset. From left to right: input image, ground truth, UNet, MicroSeg-Net, PZS-Net, nnUNet, and RaNet.

Figures 3, 4 compares the segmentation results from UNet, MicroSeg-Net, PZS-Net, nnUNet, and RaNet. While UNet serves as a strong baseline, it struggles with incomplete regions and imprecise boundaries due to its lack of spatial attention and feature refinement. MicroSeg-Net improves feature map refinement but still fails to capture complete regions due to inadequate spatial resolution preservation. PZS-Net offers better region visualization but suffers from blurry boundaries due to insufficient refinement in the decoding phase. nnUNet, though comparable to RaNet in segmentation performance, faces challenges with accurately delineating zonal boundaries. In contrast, RaNet outperforms all models, delivering sharper, more complete segmentations with well-defined boundaries. This is attributed to RaNet's architectural innovations, such as the removal of max-pooling in ResNet50, dilated convolutions to preserve spatial integrity, MSAF for focusing on relevant regions, and FFM for refining the final segmentation. These enhancements make RaNet particularly effective in complex medical image segmentation tasks, ensuring high accuracy and precise boundary delineation.

## 6 Discussion

This study introduces RaNet, a novel model for prostate segmentation in MRI images, which outperforms traditional and state-of-the-art segmentation models, including UNet, MicroSeg-Net, PZS-Net, and nnUNet. The architecture of RaNet integrates several innovations, such as the DCNet encoder, MSAF, and FFM, which collectively enhance segmentation performance. RaNet achieved a DSC of 98.61% on the PROMISE12 dataset and 96.57% on the ProstateX dataset, demonstrating superior accuracy compared to the existing models. The DCNet encoder plays a crucial role in preserving spatial resolution by eliminating the initial max-pooling layer and using dilated convolutions to expand the receptive field. These modifications help RaNet capture both fine details and larger contextual features, crucial for accurate prostate segmentation. MSAF, with its multi-scale feature fusion mechanism, refines the model's ability to focus on relevant regions at multiple scales, further improving segmentation accuracy. Finally, the FFM consolidates features from various decoder layers using bilinear upsampling, ensuring robust and precise segmentation output by maintaining both spatial and semantic information. RaNet's performance was compared with other state-of-the-art models, including nnUNet, which is known for its adaptive design and strong performance across various datasets. While nnUNet performed well, RaNet outperformed it on the ProstateX dataset, achieving a DSC improvement of 0.75% and an IoU improvement of 1.09%. This superior performance can be attributed to RaNet's architectural enhancements, such as dilated convolutions and attention mechanisms, which enable more precise feature retention and better boundary delineation, particularly for the complex prostate boundaries.

## 7 Conclusion

RaNet represents a significant advancement in prostate segmentation, surpassing existing models in both accuracy and efficiency. The integration of a deep ResNet-based encoder, attention mechanisms, and optimized pooling strategies enables RaNet to achieve superior performance, even with smaller datasets. These results suggest RaNet could be a valuable tool in clinical applications for prostate cancer diagnosis, improving diagnostic accuracy and treatment planning. Accurate segmentation of prostate regions is essential for clinicians to develop effective treatment strategies, as precise delineation directly influences outcomes. RaNet's high accuracy, even when trained on limited data, makes it a promising solution for real-world medical scenarios with scarce annotated data. Despite its advantages, RaNet faces challenges in high-resolution image processing and requires further optimization for computational efficiency. Additionally, extensive validation in clinical settings is needed to ensure its generalizability across diverse patient populations and imaging protocols. While RaNet performs well with smaller datasets, its computational demand could be a limitation for widespread use in real-time clinical scenarios. Future work will focus on improving RaNet's computational efficiency, particularly for high-resolution medical images, ensuring that inference speed is optimized while maintaining accuracy for real-time clinical use. Additionally, expanding its application to other medical imaging tasks, such as tumor detection and organ segmentation, will enhance its clinical relevance and solidify its role in medical image analysis and clinical decision-making.

## Data Availability

Publicly available datasets were analyzed in this study. This data can be found here: https://promise12.grand-challenge.org/; https://www.cancerimagingarchive.net/collection/prostatex/.
